# Pseudoaglycone of Spinosyn A

**DOI:** 10.1107/S1600536812028851

**Published:** 2012-07-18

**Authors:** Hongxin Chai, Mingxing Liu, Qi Zhang, Daxin Shi, Jiarong Li

**Affiliations:** a School of Chemical Engineering and Environment, Beijing Institute of Technology, Beijing 100081, People’s Republic of China

## Abstract

The title compound [systematic name: 9-ethyl-13-hy­droxy-14-methyl-2-(3,4,5-trimeth­oxy-6-methyl­tetra­hydro-2*H*-pyran-2-yl­oxy)-3,3a,5b,6,9,10,11,12,13,14,16a,16b-dodeca­hydro-1*H*-*as*-indaceno[3,2-*d*][1]oxacyclo­dodecine-7,15(2*H*,5a*H*)-dione], C_33_H_50_O_9_, was obtained by hydrolysis of Spinosyn A. The fused cyclo­pentene ring adopts a twisted conformation, while the fused cyclo­hexene and cyclo­pentane rings are in envelope conformations with the same C atom at the flaps. In the crystal, mol­ecules are linked by O—H⋯O and C—H⋯O hydrogen bonds into a layer parallel to the *ab* plane.

## Related literature

For the insecticidal activity and research background of Spinosyn, see: Sparks *et al.* (2008 ▶); Thompson *et al.* (2000 ▶); Salgado *et al.* (1998 ▶). For the structure of Spinosyn A, see: Evans & Black (1993 ▶).
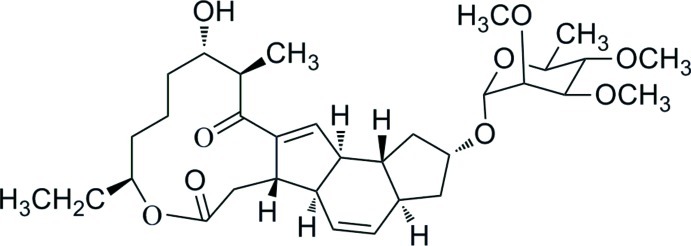


## Experimental

### 

#### Crystal data

C_33_H_50_O_9_
*M* *_r_* = 590.73
Orthorhombic, 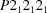

*a* = 8.7776 (15) Å
*b* = 8.7959 (15) Å
*c* = 41.737 (7) Å
*V* = 3222.4 (10) Å^3^
*Z* = 4
Mo *K*α radiation
μ = 0.09 mm^−1^
*T* = 153 K
0.34 × 0.27 × 0.08 mm

#### Data collection

Rigaku AFC10/Saturn724+ diffractometer
25322 measured reflections
4876 independent reflections
4194 reflections with *I* > 2σ(*I*)
*R*_int_ = 0.047

#### Refinement

*R*[*F* ^2^ > 2σ(*F* ^2^)] = 0.053
*wR*(*F* ^2^) = 0.134
*S* = 1.00
4876 reflections
389 parameters
H atoms treated by a mixture of independent and constrained refinement
Δρ_max_ = 0.75 e Å^−3^
Δρ_min_ = −0.22 e Å^−3^

### 

Data collection: *CrystalClear* (Rigaku, 2008 ▶); cell refinement: *CrystalClear*; data reduction: *CrystalClear*; program(s) used to solve structure: *SHELXS97* (Sheldrick, 2008 ▶); program(s) used to refine structure: *SHELXL97* (Sheldrick, 2008 ▶); molecular graphics: *CrystalStructure* (Rigaku/MSC, 2009 ▶); software used to prepare material for publication: *CrystalStructure*.

## Supplementary Material

Crystal structure: contains datablock(s) I, global. DOI: 10.1107/S1600536812028851/is5144sup1.cif

Structure factors: contains datablock(s) I. DOI: 10.1107/S1600536812028851/is5144Isup2.hkl

Additional supplementary materials:  crystallographic information; 3D view; checkCIF report

## Figures and Tables

**Table 1:** Hydrogen-bond geometry (Å, °)

*D*—H⋯*A*	*D*—H	H⋯*A*	*D*⋯*A*	*D*—H⋯*A*
O16—H16*O*⋯O18^i^	0.92 (4)	1.96 (3)	2.840 (3)	160 (3)
C3—H3*B*⋯O10^ii^	0.99	2.47	3.310 (3)	142

Symmetry codes: (i) 
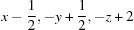
; (ii) 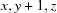.

**Fig. 1.**
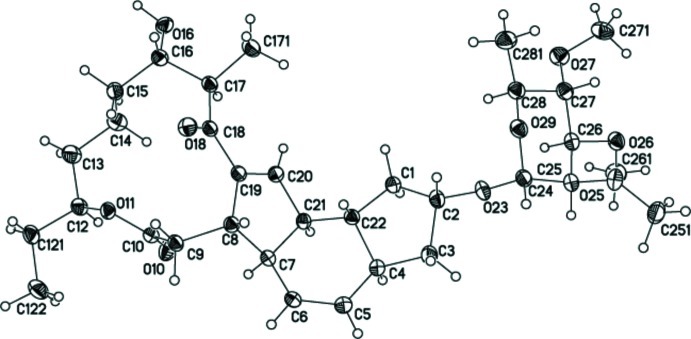
The molecular structure of the title compound with the atom numbering scheme. Displacement ellipsoids are drawn at the 30% probability level.

## supplementary crystallographic information

### Comment

Spinosyns, a novel class of green pesticides, were characterized with high efficiency, fast degradation (Sparks *et al.*, 2008), low toxicity (Thompson *et al.*, 2000) and safety to environment (Salgado *et al.*, 1998). Inspired by the high and broad insecticidal activity of Spinosyns and continuing our interest in its structure modification, we obtained pseudoaglycone of Spinosyn A from hydrolysis of the amino sugar forosamine. Here, we report the crystal structure of the title compound (Fig. 1).

### Experimental

A solution of 2.0 g Spinosyn A (purchased from SHANGHAI HOHANCE GROUP, 98%) and 5eq H_2_SO_4_ was heated to 80 °C in ethanol (20 ml) for 2 h. The reaction mixture was cooled to room temperature and then filtered to give the title compound. The product was recrystallizated from ethanol to give colourless crystalline powder (*m.p.* 441–443 K).

### Refinement

C-bound H atoms were included in a riding model approximation with C—H distances 0.95–1.00 Å, and with *U*_iso_(H) = 1.2*U*_eq_(C). The H atom of OH group was located in a difference Fourrier map and refined freely [O—H = 0.92 (4) Å]. The absolute configuration was determined according to the structure of Spinosyn A (Evans & Black, 1993).

### Crystal data

**Table:**

C_33_H_50_O_9_	*F*(000) = 1280
*M**_r_* = 590.73	*D*_x_ = 1.218 Mg m^−^^3^
Orthorhombic, *P*2_1_2_1_2_1_	Mo *K*α radiation, λ = 0.71073 Å
Hall symbol: P 2ac 2ab	Cell parameters from 11776 reflections
*a* = 8.7776 (15) Å	θ = 2.4–29.1°
*b* = 8.7959 (15) Å	µ = 0.09 mm^−^^1^
*c* = 41.737 (7) Å	*T* = 153 K
*V* = 3222.4 (10) Å^3^	Platelet, colorless
*Z* = 4	0.34 × 0.27 × 0.08 mm

### Data collection

**Table:**

Rigaku AFC10/Saturn724+ diffractometer	4194 reflections with *I* > 2σ(*I*)
Radiation source: Rotating Anode	*R*_int_ = 0.047
Graphite monochromator	θ_max_ = 29.1°, θ_min_ = 2.4°
Detector resolution: 28.5714 pixels mm^-1^	*h* = −10→12
φ and ω scans	*k* = −11→12
25322 measured reflections	*l* = −57→43
4876 independent reflections	

### Refinement

**Table:**

Refinement on *F*^2^	Primary atom site location: structure-invariant direct methods
Least-squares matrix: full	Secondary atom site location: difference Fourier map
*R*[*F*^2^ > 2σ(*F*^2^)] = 0.053	Hydrogen site location: inferred from neighbouring sites
*wR*(*F*^2^) = 0.134	H atoms treated by a mixture of independent and constrained refinement
*S* = 1.00	*w* = 1/[σ^2^(*F*_o_^2^) + (0.0696*P*)^2^ + 0.586*P*] where *P* = (*F*_o_^2^ + 2*F*_c_^2^)/3
4876 reflections	(Δ/σ)_max_ = 0.001
389 parameters	Δρ_max_ = 0.75 e Å^−^^3^
0 restraints	Δρ_min_ = −0.22 e Å^−^^3^

### Special details

**Table:**

Experimental. Spectral data: IR (KBr): 3517, 2968, 2930, 1624, 1650, 1606, 1457, 1379, cm^-1^; MS (MALDI-TOF) m/*z*: [*M*+Na]^+^ 613.3.
Geometry. All e.s.d.'s (except the e.s.d. in the dihedral angle between two l.s. planes) are estimated using the full covariance matrix. The cell e.s.d.'s are taken into account individually in the estimation of e.s.d.'s in distances, angles and torsion angles; correlations between e.s.d.'s in cell parameters are only used when they are defined by crystal symmetry. An approximate (isotropic) treatment of cell e.s.d.'s is used for estimating e.s.d.'s involving l.s. planes.
Refinement. Refinement of *F*^2^ against ALL reflections. The weighted *R*-factor *wR* and goodness of fit *S* are based on *F*^2^, conventional *R*-factors *R* are based on *F*, with *F* set to zero for negative *F*^2^. The threshold expression of *F*^2^ > σ(*F*^2^) is used only for calculating *R*-factors(gt) *etc*. and is not relevant to the choice of reflections for refinement. *R*-factors based on *F*^2^ are statistically about twice as large as those based on *F*, and *R*- factors based on ALL data will be even larger.

### Fractional atomic coordinates and isotropic or equivalent isotropic displacement parameters (Å^2^)

**Table:**

	*x*	*y*	*z*	*U*_iso_*/*U*_eq_	
O10	0.5636 (3)	0.0686 (2)	0.89590 (4)	0.0513 (6)	
O11	0.5456 (2)	0.04001 (18)	0.94906 (4)	0.0318 (4)	
O16	−0.0506 (2)	0.1699 (2)	0.96650 (5)	0.0425 (5)	
O18	0.3702 (2)	0.4082 (2)	0.96960 (4)	0.0375 (4)	
O23	0.2851 (2)	0.94913 (19)	0.81098 (4)	0.0368 (4)	
O25	0.2547 (2)	1.3408 (2)	0.78898 (4)	0.0412 (5)	
O26	0.0084 (3)	1.2315 (2)	0.75097 (5)	0.0403 (4)	
O27	−0.1883 (2)	1.1350 (2)	0.80154 (5)	0.0455 (5)	
O29	0.1799 (2)	1.1452 (2)	0.84138 (4)	0.0357 (4)	
C1	0.3003 (3)	0.7119 (3)	0.84098 (6)	0.0350 (5)	
H1A	0.2102	0.7109	0.8553	0.042*	
H1B	0.2732	0.6649	0.8202	0.042*	
C2	0.3627 (3)	0.8721 (3)	0.83662 (6)	0.0340 (5)	
H2	0.3465	0.9303	0.8569	0.041*	
C3	0.5355 (3)	0.8560 (3)	0.83072 (7)	0.0386 (6)	
H3A	0.5623	0.8882	0.8087	0.046*	
H3B	0.5940	0.9182	0.8462	0.046*	
C4	0.5683 (3)	0.6857 (3)	0.83553 (6)	0.0319 (5)	
H4	0.5598	0.6341	0.8143	0.038*	
C5	0.7166 (3)	0.6402 (3)	0.85043 (7)	0.0386 (6)	
H5	0.8054	0.6996	0.8468	0.046*	
C6	0.7238 (3)	0.5161 (3)	0.86879 (6)	0.0358 (5)	
H6	0.8196	0.4910	0.8779	0.043*	
C7	0.5914 (3)	0.4131 (3)	0.87605 (6)	0.0313 (5)	
H7	0.6175	0.3089	0.8681	0.038*	
C8	0.5579 (3)	0.4018 (3)	0.91260 (6)	0.0295 (5)	
H8	0.5880	0.5004	0.9227	0.035*	
C9	0.6420 (3)	0.2733 (3)	0.93019 (6)	0.0307 (5)	
H9A	0.7513	0.2766	0.9243	0.037*	
H9B	0.6345	0.2907	0.9536	0.037*	
C10	0.5798 (3)	0.1174 (3)	0.92253 (6)	0.0315 (5)	
C12	0.5017 (3)	−0.1199 (3)	0.94565 (7)	0.0357 (5)	
H12	0.4991	−0.1471	0.9224	0.043*	
C13	0.3448 (4)	−0.1438 (3)	0.95999 (9)	0.0489 (7)	
H13A	0.3546	−0.1466	0.9836	0.059*	
H13B	0.3063	−0.2442	0.9530	0.059*	
C14	0.2249 (4)	−0.0210 (3)	0.95098 (8)	0.0449 (7)	
H14A	0.2574	0.0328	0.9313	0.054*	
H14B	0.1254	−0.0701	0.9467	0.054*	
C15	0.2085 (3)	0.0922 (3)	0.97844 (7)	0.0381 (6)	
H15A	0.1735	0.0361	0.9976	0.046*	
H15B	0.3107	0.1338	0.9834	0.046*	
C16	0.1004 (3)	0.2250 (3)	0.97290 (6)	0.0355 (5)	
H16	0.0975	0.2888	0.9927	0.043*	
C17	0.1391 (3)	0.3263 (3)	0.94464 (6)	0.0313 (5)	
H17	0.1169	0.2696	0.9244	0.038*	
C18	0.3045 (3)	0.3737 (3)	0.94445 (6)	0.0293 (5)	
C19	0.3866 (3)	0.3896 (3)	0.91396 (6)	0.0293 (5)	
C20	0.3231 (3)	0.4125 (3)	0.88526 (6)	0.0311 (5)	
H20	0.2176	0.3998	0.8809	0.037*	
C21	0.4377 (3)	0.4603 (3)	0.86074 (6)	0.0297 (5)	
H21	0.4207	0.4068	0.8399	0.036*	
C22	0.4360 (3)	0.6331 (3)	0.85635 (6)	0.0292 (5)	
H22	0.4505	0.6794	0.8780	0.035*	
C24	0.2789 (3)	1.1066 (3)	0.81606 (6)	0.0336 (5)	
H24	0.3835	1.1431	0.8216	0.040*	
C25	0.2305 (3)	1.1817 (3)	0.78489 (6)	0.0342 (5)	
H25	0.2956	1.1435	0.7670	0.041*	
C26	0.0631 (3)	1.1468 (3)	0.77771 (6)	0.0334 (5)	
H26	0.0529	1.0360	0.7729	0.040*	
C27	−0.0359 (3)	1.1844 (3)	0.80648 (6)	0.0351 (5)	
H27	−0.0345	1.2965	0.8104	0.042*	
C28	0.0250 (3)	1.1013 (3)	0.83596 (6)	0.0367 (5)	
H28	0.0209	0.9892	0.8320	0.044*	
C121	0.6240 (4)	−0.2127 (3)	0.96221 (7)	0.0422 (6)	
H12A	0.6335	−0.1777	0.9847	0.051*	
H12B	0.5920	−0.3206	0.9627	0.051*	
C122	0.7782 (4)	−0.2017 (4)	0.94616 (9)	0.0537 (8)	
H12C	0.8121	−0.0955	0.9461	0.064*	
H12D	0.7703	−0.2382	0.9240	0.064*	
H12E	0.8519	−0.2641	0.9579	0.064*	
C171	0.0413 (3)	0.4720 (3)	0.94530 (8)	0.0438 (6)	
H17A	0.0594	0.5308	0.9257	0.053*	
H17B	0.0690	0.5333	0.9640	0.053*	
H17C	−0.0667	0.4444	0.9466	0.053*	
C251	0.3124 (4)	1.4170 (4)	0.76164 (8)	0.0516 (8)	
H25A	0.2365	1.4139	0.7444	0.062*	
H25B	0.4061	1.3668	0.7545	0.062*	
H25C	0.3347	1.5230	0.7671	0.062*	
C261	0.0420 (4)	1.1631 (4)	0.72113 (7)	0.0528 (8)	
H26A	−0.0006	1.0601	0.7206	0.063*	
H26B	0.1527	1.1580	0.7183	0.063*	
H26C	−0.0029	1.2237	0.7038	0.063*	
C271	−0.2890 (4)	1.2497 (4)	0.79068 (8)	0.0524 (8)	
H27A	−0.2558	1.2859	0.7696	0.063*	
H27B	−0.2886	1.3346	0.8059	0.063*	
H27C	−0.3922	1.2080	0.7890	0.063*	
C281	−0.0613 (4)	1.1369 (4)	0.86623 (7)	0.0502 (7)	
H28A	−0.0567	1.2464	0.8704	0.060*	
H28B	−0.0154	1.0817	0.8842	0.060*	
H28C	−0.1678	1.1056	0.8638	0.060*	
H16O	−0.083 (4)	0.123 (4)	0.9849 (8)	0.057 (10)*	

### Atomic displacement parameters (Å^2^)

**Table:**

	*U*^11^	*U*^22^	*U*^33^	*U*^12^	*U*^13^	*U*^23^
O10	0.0789 (16)	0.0422 (10)	0.0329 (10)	−0.0082 (11)	0.0023 (11)	−0.0019 (8)
O11	0.0363 (9)	0.0259 (7)	0.0333 (9)	0.0026 (7)	0.0007 (7)	0.0025 (6)
O16	0.0279 (9)	0.0581 (12)	0.0415 (11)	−0.0038 (9)	0.0016 (8)	0.0115 (9)
O18	0.0358 (10)	0.0461 (10)	0.0307 (9)	−0.0012 (8)	−0.0039 (8)	−0.0016 (7)
O23	0.0478 (11)	0.0299 (8)	0.0327 (9)	0.0108 (8)	−0.0055 (8)	0.0000 (7)
O25	0.0510 (12)	0.0312 (9)	0.0415 (10)	−0.0039 (8)	−0.0036 (9)	0.0063 (7)
O26	0.0445 (10)	0.0424 (10)	0.0339 (9)	0.0078 (8)	−0.0048 (8)	0.0037 (8)
O27	0.0357 (11)	0.0481 (11)	0.0527 (12)	−0.0028 (9)	−0.0035 (9)	−0.0016 (9)
O29	0.0401 (10)	0.0347 (9)	0.0324 (9)	0.0062 (8)	−0.0021 (8)	−0.0028 (7)
C1	0.0333 (13)	0.0378 (13)	0.0340 (13)	0.0042 (10)	−0.0005 (11)	0.0060 (10)
C2	0.0408 (14)	0.0318 (12)	0.0295 (12)	0.0066 (10)	−0.0033 (11)	0.0012 (9)
C3	0.0407 (15)	0.0314 (12)	0.0438 (14)	0.0002 (11)	−0.0017 (12)	0.0052 (10)
C4	0.0322 (12)	0.0308 (11)	0.0328 (12)	0.0011 (10)	0.0000 (10)	0.0028 (9)
C5	0.0310 (13)	0.0403 (13)	0.0445 (14)	−0.0004 (11)	0.0022 (11)	0.0069 (11)
C6	0.0256 (12)	0.0418 (13)	0.0399 (13)	0.0040 (10)	−0.0017 (10)	0.0072 (10)
C7	0.0297 (12)	0.0309 (11)	0.0332 (12)	0.0047 (9)	−0.0002 (10)	0.0033 (9)
C8	0.0288 (11)	0.0290 (10)	0.0307 (11)	−0.0001 (9)	−0.0013 (10)	0.0007 (9)
C9	0.0269 (11)	0.0314 (11)	0.0339 (12)	0.0021 (9)	−0.0040 (10)	0.0027 (9)
C10	0.0292 (12)	0.0329 (11)	0.0324 (12)	0.0035 (10)	0.0013 (10)	0.0007 (9)
C12	0.0391 (14)	0.0241 (11)	0.0439 (14)	0.0040 (10)	0.0016 (11)	−0.0024 (10)
C13	0.0447 (16)	0.0284 (12)	0.074 (2)	0.0017 (12)	0.0100 (15)	0.0042 (13)
C14	0.0382 (15)	0.0390 (14)	0.0574 (18)	−0.0019 (12)	0.0014 (13)	−0.0047 (12)
C15	0.0345 (13)	0.0362 (12)	0.0435 (14)	0.0004 (11)	−0.0013 (12)	0.0068 (10)
C16	0.0293 (12)	0.0416 (13)	0.0356 (13)	0.0024 (10)	−0.0020 (10)	0.0018 (10)
C17	0.0272 (12)	0.0341 (12)	0.0328 (12)	0.0036 (9)	−0.0035 (10)	0.0010 (10)
C18	0.0272 (11)	0.0306 (11)	0.0302 (11)	0.0042 (9)	−0.0023 (9)	0.0012 (9)
C19	0.0276 (11)	0.0286 (11)	0.0316 (11)	0.0025 (9)	−0.0009 (9)	0.0009 (9)
C20	0.0293 (12)	0.0294 (11)	0.0346 (12)	−0.0019 (9)	−0.0017 (10)	0.0007 (9)
C21	0.0306 (12)	0.0305 (10)	0.0280 (11)	0.0007 (10)	−0.0025 (9)	0.0017 (9)
C22	0.0289 (11)	0.0314 (11)	0.0274 (11)	0.0026 (9)	−0.0010 (9)	−0.0003 (8)
C24	0.0348 (13)	0.0322 (12)	0.0338 (12)	0.0057 (10)	−0.0004 (10)	0.0015 (9)
C25	0.0376 (14)	0.0284 (11)	0.0366 (13)	0.0025 (10)	−0.0015 (11)	0.0026 (9)
C26	0.0389 (13)	0.0292 (11)	0.0321 (12)	0.0028 (10)	−0.0035 (11)	0.0003 (9)
C27	0.0334 (13)	0.0336 (12)	0.0383 (13)	0.0011 (10)	−0.0042 (11)	−0.0012 (10)
C28	0.0393 (14)	0.0359 (12)	0.0350 (13)	0.0037 (11)	0.0009 (11)	−0.0007 (10)
C121	0.0507 (17)	0.0298 (12)	0.0460 (15)	0.0121 (11)	0.0019 (13)	0.0022 (11)
C122	0.0465 (18)	0.0461 (16)	0.069 (2)	0.0170 (14)	0.0014 (16)	0.0023 (15)
C171	0.0347 (14)	0.0420 (14)	0.0547 (16)	0.0071 (12)	−0.0002 (13)	0.0040 (13)
C251	0.0525 (19)	0.0458 (16)	0.0565 (18)	−0.0060 (14)	−0.0004 (15)	0.0164 (13)
C261	0.060 (2)	0.0646 (19)	0.0341 (14)	0.0067 (17)	−0.0037 (14)	−0.0034 (13)
C271	0.0355 (15)	0.069 (2)	0.0528 (18)	0.0098 (15)	−0.0034 (14)	−0.0046 (15)
C281	0.0521 (18)	0.0603 (18)	0.0383 (15)	0.0103 (16)	0.0090 (14)	−0.0005 (13)

### Geometric parameters (Å, º)

**Table:**

O10—C10	1.200 (3)	C14—H14A	0.9900
O11—C10	1.334 (3)	C14—H14B	0.9900
O11—C12	1.465 (3)	C15—C16	1.523 (4)
O16—C16	1.436 (3)	C15—H15A	0.9900
O16—H16O	0.92 (4)	C15—H15B	0.9900
O18—C18	1.236 (3)	C16—C17	1.517 (3)
O23—C24	1.402 (3)	C16—H16	1.0000
O23—C2	1.438 (3)	C17—C18	1.510 (3)
O25—C251	1.417 (3)	C17—C171	1.543 (4)
O25—C25	1.425 (3)	C17—H17	1.0000
O26—C261	1.414 (3)	C18—C19	1.469 (3)
O26—C26	1.425 (3)	C19—C20	1.336 (3)
O27—C271	1.416 (4)	C20—C21	1.496 (3)
O27—C27	1.421 (3)	C20—H20	0.9500
O29—C24	1.410 (3)	C21—C22	1.531 (3)
O29—C28	1.432 (3)	C21—H21	1.0000
C1—C22	1.520 (3)	C22—H22	1.0000
C1—C2	1.523 (4)	C24—C25	1.519 (3)
C1—H1A	0.9900	C24—H24	1.0000
C1—H1B	0.9900	C25—C26	1.531 (4)
C2—C3	1.543 (4)	C25—H25	1.0000
C2—H2	1.0000	C26—C27	1.518 (4)
C3—C4	1.538 (3)	C26—H26	1.0000
C3—H3A	0.9900	C27—C28	1.527 (4)
C3—H3B	0.9900	C27—H27	1.0000
C4—C5	1.497 (4)	C28—C281	1.506 (4)
C4—C22	1.522 (3)	C28—H28	1.0000
C4—H4	1.0000	C121—C122	1.513 (5)
C5—C6	1.335 (4)	C121—H12A	0.9900
C5—H5	0.9500	C121—H12B	0.9900
C6—C7	1.505 (4)	C122—H12C	0.9800
C6—H6	0.9500	C122—H12D	0.9800
C7—C21	1.550 (3)	C122—H12E	0.9800
C7—C8	1.557 (3)	C171—H17A	0.9800
C7—H7	1.0000	C171—H17B	0.9800
C8—C19	1.509 (3)	C171—H17C	0.9800
C8—C9	1.537 (3)	C251—H25A	0.9800
C8—H8	1.0000	C251—H25B	0.9800
C9—C10	1.511 (3)	C251—H25C	0.9800
C9—H9A	0.9900	C261—H26A	0.9800
C9—H9B	0.9900	C261—H26B	0.9800
C12—C121	1.516 (4)	C261—H26C	0.9800
C12—C13	1.516 (4)	C271—H27A	0.9800
C12—H12	1.0000	C271—H27B	0.9800
C13—C14	1.554 (4)	C271—H27C	0.9800
C13—H13A	0.9900	C281—H28A	0.9800
C13—H13B	0.9900	C281—H28B	0.9800
C14—C15	1.525 (4)	C281—H28C	0.9800
			
C10—O11—C12	117.9 (2)	O18—C18—C19	118.9 (2)
C16—O16—H16O	106 (2)	O18—C18—C17	120.8 (2)
C24—O23—C2	111.83 (19)	C19—C18—C17	120.1 (2)
C251—O25—C25	114.9 (2)	C20—C19—C18	125.9 (2)
C261—O26—C26	113.4 (2)	C20—C19—C8	111.8 (2)
C271—O27—C27	114.6 (2)	C18—C19—C8	121.9 (2)
C24—O29—C28	113.7 (2)	C19—C20—C21	112.0 (2)
C22—C1—C2	101.0 (2)	C19—C20—H20	124.0
C22—C1—H1A	111.6	C21—C20—H20	124.0
C2—C1—H1A	111.6	C20—C21—C22	110.8 (2)
C22—C1—H1B	111.6	C20—C21—C7	103.17 (18)
C2—C1—H1B	111.6	C22—C21—C7	108.9 (2)
H1A—C1—H1B	109.4	C20—C21—H21	111.2
O23—C2—C1	110.8 (2)	C22—C21—H21	111.2
O23—C2—C3	112.9 (2)	C7—C21—H21	111.2
C1—C2—C3	106.7 (2)	C1—C22—C4	102.61 (19)
O23—C2—H2	108.8	C1—C22—C21	120.7 (2)
C1—C2—H2	108.8	C4—C22—C21	111.3 (2)
C3—C2—H2	108.8	C1—C22—H22	107.2
C4—C3—C2	104.6 (2)	C4—C22—H22	107.2
C4—C3—H3A	110.8	C21—C22—H22	107.2
C2—C3—H3A	110.8	O23—C24—O29	112.0 (2)
C4—C3—H3B	110.8	O23—C24—C25	108.1 (2)
C2—C3—H3B	110.8	O29—C24—C25	111.4 (2)
H3A—C3—H3B	108.9	O23—C24—H24	108.4
C5—C4—C22	110.2 (2)	O29—C24—H24	108.4
C5—C4—C3	118.5 (2)	C25—C24—H24	108.4
C22—C4—C3	103.2 (2)	O25—C25—C24	106.4 (2)
C5—C4—H4	108.2	O25—C25—C26	111.3 (2)
C22—C4—H4	108.2	C24—C25—C26	110.4 (2)
C3—C4—H4	108.2	O25—C25—H25	109.5
C6—C5—C4	119.9 (2)	C24—C25—H25	109.5
C6—C5—H5	120.1	C26—C25—H25	109.5
C4—C5—H5	120.1	O26—C26—C27	108.2 (2)
C5—C6—C7	124.9 (2)	O26—C26—C25	111.8 (2)
C5—C6—H6	117.6	C27—C26—C25	110.5 (2)
C7—C6—H6	117.6	O26—C26—H26	108.7
C6—C7—C21	115.32 (19)	C27—C26—H26	108.7
C6—C7—C8	112.4 (2)	C25—C26—H26	108.7
C21—C7—C8	104.87 (19)	O27—C27—C26	110.9 (2)
C6—C7—H7	108.0	O27—C27—C28	107.5 (2)
C21—C7—H7	108.0	C26—C27—C28	109.4 (2)
C8—C7—H7	108.0	O27—C27—H27	109.7
C19—C8—C9	114.1 (2)	C26—C27—H27	109.7
C19—C8—C7	103.3 (2)	C28—C27—H27	109.7
C9—C8—C7	115.1 (2)	O29—C28—C281	106.8 (2)
C19—C8—H8	108.0	O29—C28—C27	109.3 (2)
C9—C8—H8	108.0	C281—C28—C27	113.6 (2)
C7—C8—H8	108.0	O29—C28—H28	109.0
C10—C9—C8	113.1 (2)	C281—C28—H28	109.0
C10—C9—H9A	109.0	C27—C28—H28	109.0
C8—C9—H9A	109.0	C122—C121—C12	113.4 (2)
C10—C9—H9B	109.0	C122—C121—H12A	108.9
C8—C9—H9B	109.0	C12—C121—H12A	108.9
H9A—C9—H9B	107.8	C122—C121—H12B	108.9
O10—C10—O11	124.0 (2)	C12—C121—H12B	108.9
O10—C10—C9	124.3 (2)	H12A—C121—H12B	107.7
O11—C10—C9	111.6 (2)	C121—C122—H12C	109.5
O11—C12—C121	106.6 (2)	C121—C122—H12D	109.5
O11—C12—C13	109.5 (2)	H12C—C122—H12D	109.5
C121—C12—C13	112.9 (2)	C121—C122—H12E	109.5
O11—C12—H12	109.3	H12C—C122—H12E	109.5
C121—C12—H12	109.3	H12D—C122—H12E	109.5
C13—C12—H12	109.3	C17—C171—H17A	109.5
C12—C13—C14	115.1 (2)	C17—C171—H17B	109.5
C12—C13—H13A	108.5	H17A—C171—H17B	109.5
C14—C13—H13A	108.5	C17—C171—H17C	109.5
C12—C13—H13B	108.5	H17A—C171—H17C	109.5
C14—C13—H13B	108.5	H17B—C171—H17C	109.5
H13A—C13—H13B	107.5	O25—C251—H25A	109.5
C15—C14—C13	109.6 (3)	O25—C251—H25B	109.5
C15—C14—H14A	109.8	H25A—C251—H25B	109.5
C13—C14—H14A	109.8	O25—C251—H25C	109.5
C15—C14—H14B	109.8	H25A—C251—H25C	109.5
C13—C14—H14B	109.8	H25B—C251—H25C	109.5
H14A—C14—H14B	108.2	O26—C261—H26A	109.5
C16—C15—C14	116.5 (2)	O26—C261—H26B	109.5
C16—C15—H15A	108.2	H26A—C261—H26B	109.5
C14—C15—H15A	108.2	O26—C261—H26C	109.5
C16—C15—H15B	108.2	H26A—C261—H26C	109.5
C14—C15—H15B	108.2	H26B—C261—H26C	109.5
H15A—C15—H15B	107.3	O27—C271—H27A	109.5
O16—C16—C17	105.1 (2)	O27—C271—H27B	109.5
O16—C16—C15	110.1 (2)	H27A—C271—H27B	109.5
C17—C16—C15	115.4 (2)	O27—C271—H27C	109.5
O16—C16—H16	108.7	H27A—C271—H27C	109.5
C17—C16—H16	108.7	H27B—C271—H27C	109.5
C15—C16—H16	108.7	C28—C281—H28A	109.5
C18—C17—C16	112.4 (2)	C28—C281—H28B	109.5
C18—C17—C171	107.8 (2)	H28A—C281—H28B	109.5
C16—C17—C171	110.4 (2)	C28—C281—H28C	109.5
C18—C17—H17	108.7	H28A—C281—H28C	109.5
C16—C17—H17	108.7	H28B—C281—H28C	109.5
C171—C17—H17	108.7		
			
C24—O23—C2—C1	149.8 (2)	C8—C19—C20—C21	−6.2 (3)
C24—O23—C2—C3	−90.6 (3)	C19—C20—C21—C22	−98.5 (2)
C22—C1—C2—O23	155.1 (2)	C19—C20—C21—C7	17.9 (3)
C22—C1—C2—C3	31.7 (3)	C6—C7—C21—C20	−146.0 (2)
O23—C2—C3—C4	−127.8 (2)	C8—C7—C21—C20	−21.8 (2)
C1—C2—C3—C4	−5.8 (3)	C6—C7—C21—C22	−28.2 (3)
C2—C3—C4—C5	−144.5 (2)	C8—C7—C21—C22	96.0 (2)
C2—C3—C4—C22	−22.5 (3)	C2—C1—C22—C4	−46.0 (2)
C22—C4—C5—C6	29.0 (3)	C2—C1—C22—C21	−170.4 (2)
C3—C4—C5—C6	147.4 (3)	C5—C4—C22—C1	170.4 (2)
C4—C5—C6—C7	0.3 (4)	C3—C4—C22—C1	43.0 (2)
C5—C6—C7—C21	−0.5 (4)	C5—C4—C22—C21	−59.1 (3)
C5—C6—C7—C8	−120.7 (3)	C3—C4—C22—C21	173.4 (2)
C6—C7—C8—C19	144.5 (2)	C20—C21—C22—C1	−68.8 (3)
C21—C7—C8—C19	18.5 (2)	C7—C21—C22—C1	178.4 (2)
C6—C7—C8—C9	−90.5 (3)	C20—C21—C22—C4	171.0 (2)
C21—C7—C8—C9	143.5 (2)	C7—C21—C22—C4	58.2 (3)
C19—C8—C9—C10	46.5 (3)	C2—O23—C24—O29	−70.2 (3)
C7—C8—C9—C10	−72.7 (3)	C2—O23—C24—C25	166.7 (2)
C12—O11—C10—O10	7.1 (4)	C28—O29—C24—O23	−61.6 (3)
C12—O11—C10—C9	−172.6 (2)	C28—O29—C24—C25	59.7 (3)
C8—C9—C10—O10	53.0 (4)	C251—O25—C25—C24	141.0 (2)
C8—C9—C10—O11	−127.4 (2)	C251—O25—C25—C26	−98.7 (3)
C10—O11—C12—C121	115.1 (2)	O23—C24—C25—O25	−168.6 (2)
C10—O11—C12—C13	−122.4 (3)	O29—C24—C25—O25	67.8 (3)
O11—C12—C13—C14	44.8 (3)	O23—C24—C25—C26	70.4 (3)
C121—C12—C13—C14	163.4 (3)	O29—C24—C25—C26	−53.1 (3)
C12—C13—C14—C15	−100.1 (3)	C261—O26—C26—C27	−154.1 (2)
C13—C14—C15—C16	177.2 (2)	C261—O26—C26—C25	83.9 (3)
C14—C15—C16—O16	59.5 (3)	O25—C25—C26—O26	54.3 (3)
C14—C15—C16—C17	−59.3 (3)	C24—C25—C26—O26	172.3 (2)
O16—C16—C17—C18	−170.3 (2)	O25—C25—C26—C27	−66.4 (3)
C15—C16—C17—C18	−48.8 (3)	C24—C25—C26—C27	51.6 (3)
O16—C16—C17—C171	69.2 (3)	C271—O27—C27—C26	−97.4 (3)
C15—C16—C17—C171	−169.3 (2)	C271—O27—C27—C28	143.0 (2)
C16—C17—C18—O18	−40.1 (3)	O26—C26—C27—O27	64.4 (3)
C171—C17—C18—O18	81.8 (3)	C25—C26—C27—O27	−172.8 (2)
C16—C17—C18—C19	144.2 (2)	O26—C26—C27—C28	−177.2 (2)
C171—C17—C18—C19	−93.9 (3)	C25—C26—C27—C28	−54.4 (3)
O18—C18—C19—C20	−153.7 (2)	C24—O29—C28—C281	174.7 (2)
C17—C18—C19—C20	22.1 (4)	C24—O29—C28—C27	−62.0 (3)
O18—C18—C19—C8	18.1 (4)	O27—C27—C28—O29	178.69 (19)
C17—C18—C19—C8	−166.1 (2)	C26—C27—C28—O29	58.1 (3)
C9—C8—C19—C20	−133.9 (2)	O27—C27—C28—C281	−62.2 (3)
C7—C8—C19—C20	−8.3 (3)	C26—C27—C28—C281	177.3 (2)
C9—C8—C19—C18	53.3 (3)	O11—C12—C121—C122	−65.3 (3)
C7—C8—C19—C18	178.9 (2)	C13—C12—C121—C122	174.4 (3)
C18—C19—C20—C21	166.3 (2)		

### Hydrogen-bond geometry (Å, º)

**Table:**

*D*—H···*A*	*D*—H	H···*A*	*D*···*A*	*D*—H···*A*
O16—H16*O*···O18^i^	0.92 (4)	1.96 (3)	2.840 (3)	160 (3)
C3—H3*B*···O10^ii^	0.99	2.47	3.310 (3)	142

Symmetry codes: (i) *x*−1/2, −*y*+1/2, −*z*+2; (ii) *x*, *y*+1, *z*.

## References

[bb1] Evans, D. A. & Black, W. C. (1993). *J. Am. Chem. Soc.* **115**, 4497–4513.

[bb2] Rigaku (2008). *CrystalClear* Rigaku Corporation, Tokyo, Japan.

[bb3] Rigaku/MSC (2009). *CrystalStructure* Rigaku/MSC, The Woodlands, Texas, USA.

[bb4] Salgado, V. L. (1998). *Pestic. Biochem. Physiol.* **60**, 91–102.

[bb5] Sheldrick, G. M. (2008). *Acta Cryst.* A**64**, 112–122. 10.1107/S0108767307043930 18156677

[bb6] Sparks, T. C., Crouse, G. D., Dripps, J. E., Anzeveno, P., Martynow, J., DeAmicis, C. V. & Gifford, J. (2008). *J. Comput. Aided Mol. Des.* **22**, 393–401. 10.1007/s10822-008-9205-8 18344004

[bb7] Thompson, G. D., Dutton, R. & Sparks, T. C. (2000). *Pest Manage. Sci.* **56**, 696–702.

